# *Gbx2* Plays an Essential but Transient Role in the Formation of Thalamic Nuclei

**DOI:** 10.1371/journal.pone.0047111

**Published:** 2012-10-04

**Authors:** Kairong Li, Jiaqing Zhang, James Y. H. Li

**Affiliations:** 1 Department of Genetics and Developmental Biology, University of Connecticut Health Center, Farmington, Connecticut, United States of America; 2 Biochemistry Department of Medical School, Jinan University, Guangzhou, Guangdong, People's Republic of China; Indiana University School of Medicine, United States of America

## Abstract

Unlike the laminar arrangement of neurons in the neocortex, thalamic neurons aggregate to form about dozens of nuclei, many of which make topographic connections with specific areas in the neocortex. The molecular mechanisms underlying the formation of thalamic nuclei remain largely unknown. Homeodomain transcription factor Gbx2 is specifically expressed in the developing thalamus. Deleting *Gbx2* leads to severe disruption of the histogenesis of the thalamus in mice, demonstrating an essential role of *Gbx2* in this brain structure. Using inducible genetic fate mapping, we have previously shown that the neuronal precursors for different sets of thalamic nuclei have distinctive onset and duration of *Gbx2* expression, suggesting that the dynamic expression of *Gbx2* plays an important role in the specification and differentiation of thalamic nuclei. Here, we showed that the *Gbx2* lineage exclusively gives rise to neurons but not glia in the thalamus. We performed conditional deletion to examine the temporal requirements of *Gbx2* in the developing thalamus in mice. Corresponding to the dynamic and differential expression of *Gbx2* in various thalamic nucleus groups, deleting *Gbx2* at different embryonic stages disrupts formation of distinct sets of thalamic nuclei. Interestingly, different thalamic nuclei have remarkably different requirements of *Gbx2* for the survival of thalamic neurons. Furthermore, although *Gbx2* expression persists in many thalamic nuclei until adulthood, only the initial expression of *Gbx2* following neurogenesis is crucial for the differentiation of thalamic nuclei. Our results indicate that the dynamic expression of *Gbx2* may act as an important determinant in coupling with other developmental programs to generate distinct thalamic nuclei.

## Introduction

The mammalian thalamus is composed of dozens of nuclei formed by aggregates of neurons, and each nucleus displays unique cytoarchitecture and connectivity [1]. The principal nuclei, which project topographically to specific areas of the cortex, have a primary role in processing and relaying periphery sensory input to the cortex, while other nuclei project broadly to the cortex and regulate the state of consciousness [1], [2]. Virtually all thalamic neurons are born between embryonic day (E) 10.5 and E16.5 in mice [3]. Between E14.5 and E18.5, the thalamus is gradually partitioned into discrete neuronal groups, signifying the differentiation of thalamic nuclei [1]. The individual thalamic nucleus becomes recognizable after birth in mice [1]. Currently, little is known about the molecular and cellular mechanisms that govern the specification, differentiation, and selective aggregation of thalamic neurons to form distinct nuclei.

The expression of *Gbx2* (Gastrulation brain homeobox gene 2) is initially detected in postmitotic cells of the thalamus in mouse embryos at E10.5 [4], [5], [6], [7]. Deletion of *Gbx2* results in nearly complete loss of thalamocortical projections, and severe defects in the histogenesis of the thalamus [5], [6], [8], [9]. In both mice and monkeys, *Gbx2* expression is maintained in a subset of thalamic nuclei to adult stages, suggesting that *Gbx2* has an evolutionarily conserved function in the establishment and maintenance of thalamic nuclei [10]. Using a *Gbx2^creER^* knock-in mouse line, we have previously performed genetic inducible fate mapping and demonstrated that the *Gbx2*-lineage contributes to the entire thalamic complex [6]. Significantly, different thalamic nuclei have distinct onset and different duration of *Gbx2* expression, suggesting that the dynamic change of *Gbx2* expression may control the formation of thalamic nuclei [6].

Mice deficient for *Gbx2* die at birth probably due to defects in the anterior hindbrain [11]. The neonatal lethality and the severe thalamus malformation have precluded the dissection of *Gbx2* function in different thalamic nuclei in *Gbx2* global knockout mutants. To circumvent these difficulties, we performed conditional knockout (CKO) experiments to remove *Gbx2* at different embryonic stages. These *Gbx2*-CKO mutant mice were viable, thus allowing examination of the formation of thalamic nuclei when they became clearly discernable at postnatal stages. Our study has revealed an essential but transient requirement of *Gbx2* in the formation of various thalamic nuclei.

**Figure 1:**
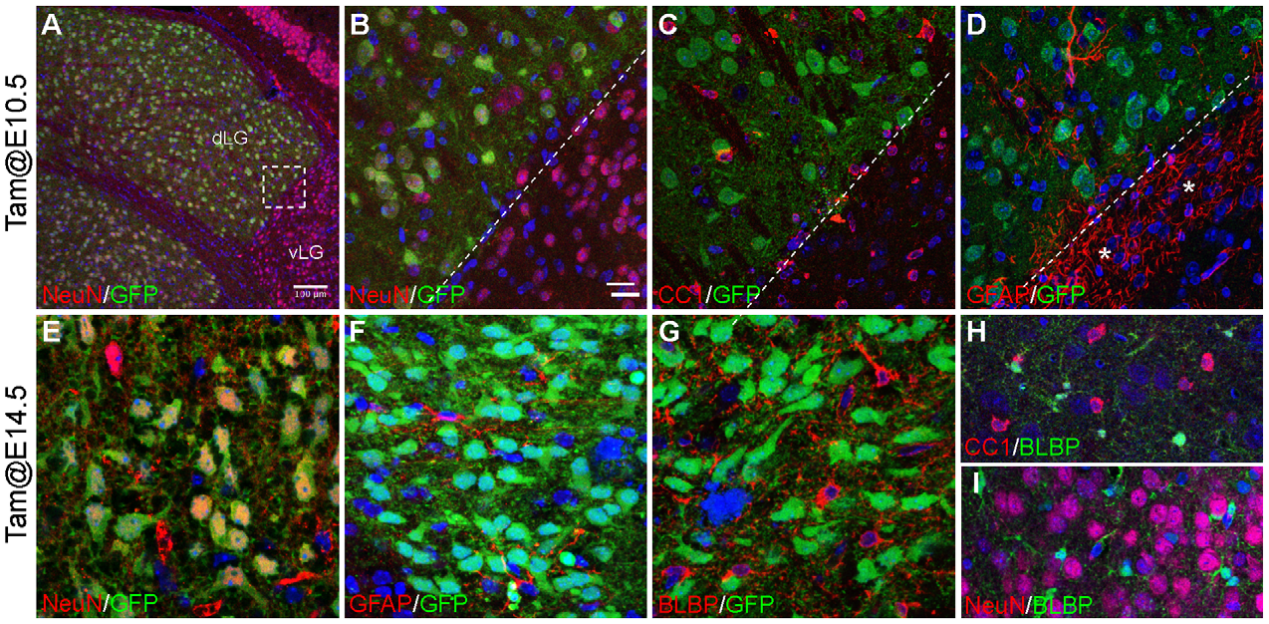
The *Gbx2*-lineage exclusively gives rise to neurons in the thalamus. (A–G) Anti-GFP staining of YFP, which is expressed from the *R26R^YFP^* locus and marks the descendants of *Gbx2*-expressing cells labeled at E10.5 (A–D) or E14.5 (F–G), and neuronal or glial markers as indicated on coronal sections of P21 *Gbx2^creER/+^; R26R^YFP^* mice. Asterisks indicate the significantly large number of GAFP^+^ cells in the IGL. (H–I) Double labeling of BLBP and NeuN or CC1 on sections of P21 thalamus. Nuclei are stained with TOPO3 (blue). Scale bars: A 100 µm; B (for B–I) 20 µm.

## Materials and Methods

### Mouse and tissue preparation

All animal procedures described herein were approved by the Animal Care Committee at the University of Connecticut Health Center. Mice were housed in a facility with a 12 h light/dark cycle and allowed free access to food and water. All mouse strains were maintained on a CD1 genetic background (Charles River Lab, Wilmington, MA). Adult mice were euthanized by carbon dioxide narcosis and followed by cervical dislocation. Neonatal mice were sacrificed by decapitation. Noon of the day on which a vaginal plug was detected was designated as E0.5 in staging of embryos. The *Gbx2^creER^* allele contains *creER-ires-EGFP* insertion at the 5′UTR so that both *creER* and *EGFP* are simultaneously expressed mimicking the endogenous *Gbx2* expression [6]. For *creER*-mediated gene deletion, *Gbx2^creER/+^* males were bred with *Gbx2^F/+^; R26R^lacZ/lacZ^* or *Gbx2^F/F^; R26R^lacZ/lacZ^* females, which carried the *Gbx2^floxed^* conditional allele (*Gbx2^F^*) [12] and a *cre* reporter, *R26R^lacZ^* [13]. Four to six milligrams of tamoxifen (Sigma, St. Louis, MO) in corn oil were administered to pregnant females by oral gavage using feeding needles. Embryos with a high percentage of recombination were selected by strong X-gal staining in the hindbrain. The recombination was subsequently confirmed by X-gal histochemistry on thalamus sections. Two additional cre-reporter lines, *R26R^YFP^* [14], and *R26R^RFP^* [15], were used in this study as indicated in the text. Immunofluorescence for GFP detected both EGFP and YFP from *Gbx2^creER^* and *R26R^YFP^* alleles, respectively, during embryonic stages. However, EGFP expression from the *Gbx2^creER^* locus became greatly reduced so that GFP antibody staining mainly detected the robust YFP signals. Embryos and mice carrying the *Gbx2^creER^* allele were identified by EGFP fluorescence in the hindbrain and spinal cord. Other alleles were determined by PCR analysis of tail DNA as described previously [12], [13].

**Figure 2:**
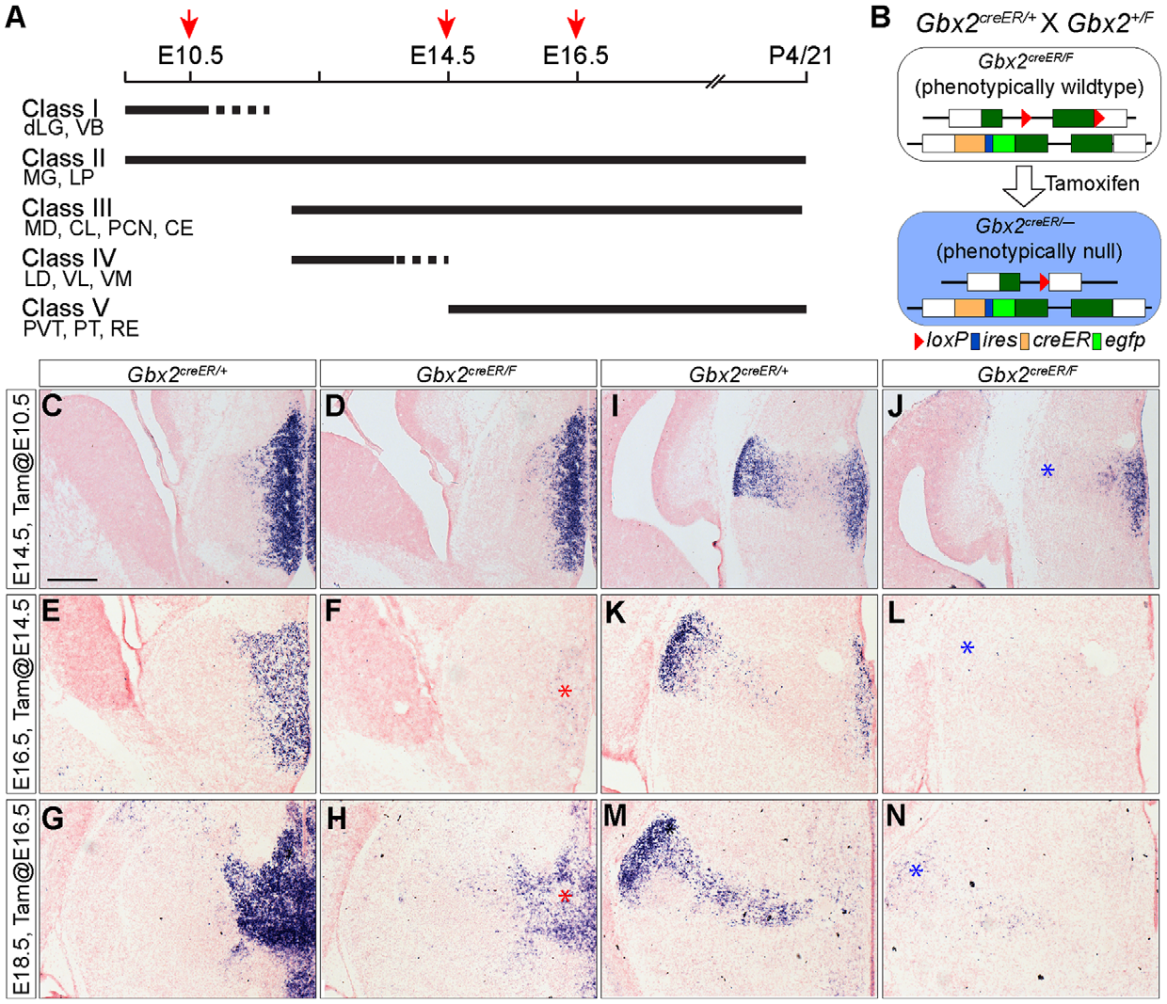
Conditional deletion of *Gbx2* at different embryonic stages. (A) Diagram of temporal cohorts of *Gbx2*-expressing cells giving rise to different groups of thalamic nuclei. *Gbx2* was deleted following administration of tamoxifen at E10.5, E14.5, or E16.5, as indicated by red arrows and the histology of thalamic nuclei was examined at P4 or P21 (Fig. 3 and 4). (B) Schematic representation of breeding scheme and “self-deletion” of *Gbx2* by creER-mediated recombination in *Gbx2^creER/F^* embryos. (C–N) ISH for *Gbx2* on coronal sections corresponding to the rostral (C–H) and caudal level (I–N) of the thalamus in control and *Gbx2*-CKO embryos between E14.5 and E18.5. The age of embryos and the stage of tamoxifen administration are indicated to the left. The RNA probe recognizes the *Gbx2* sequence that will be deleted by creER-mediated recombination. Note that the *Gbx2* transcripts are missing in the presumptive MG (red asterisk) following tamoxifen administration at E10.5, E14.5, or E16.5. By contrast, *Gbx2* expression in the rostral-medial thalamus (blue asterisk) is mostly unaffected by deletion at E10.5. Abbreviations of thalamic nuclei are listed in table I. Scale bar in A: 250 µm (for A–L); 237 µm (for G–N).

**Table 1:** Summary of defects of individual nuclei with *Gbx2* deletion at different embryonic stages.

	Thalamus nuclei	Abbreviations	Stage of *Gbx2* deletion			Groups based on fate mapping
			E10.5	E14.5	E16.5	
Anterior group	anterodorsal nucleus	AD	−	−	−	III
	anteromedial nucleus	AM	−	+	−	V
	anterioventral nucleus	AV	+	+	−	IV
	lateral dorsal nucleus	LD	−	+	−	IV
Medial group	medial dorsal nucleus	MD	−	+	−	III
	paraventricular nucleus	PVT	−	+*	−	V
	paratenial nucleus	PT	−	+*	−	V
	reunions nucleus	RE	−	+*	−	V
Intralaminar group	central medial nucleus	CM	−	+	−	III
	central lateral nucleus	CL	−	+*	−	III
	paracentral nucleus	PCN	−	+	−	III
	parafasicular nucleus rhomboid nucleus	PF RH	− −	+ +	− −	IV IV
Ventral nuclei	ventral lateral nucleus ventromedial nucleus ventrobasal nucleus	VL VM VB	+ + +	+ – −	− − −	IV IV I
Posterior group	lateral posterior nucleus	LP	+*	−	−	II
	posterior thalamic nucleus	PO	+	+*	−	IV
Lateral & medial geniculate complex	dorsal nucleus of lateral geniculate body	dLG	+	−	−	I
	principle nucleus of the medial geniculate body	MG	+*	−	−	II

−: no noticeable defect.

+: abnormal formation (some of the data were not shown in the figures).

+*: cell death.

Nomenclature and classification of thalamic nuclei follow Caviness and Frost [28], Jones [1] and Alan reference atlas (http://mouse.brain-map.org/static/atlas).

Embryonic mouse brains were dissected in cold phosphate buffered saline (PBS) and fixed in 4% paraformaldehyde (PFA) for 40 minutes. To analyze postnatal brains, mice were anesthetized by intraperitoneal injection of 2,2-tribromoethanol and followed by transcardiac perfusion with PBS and 4% PFA, and the dissected brains were fixed in 4% PFA for 3 hours. Brains were cryoprotected in 30% sucrose in PBS, frozen in OCT freezing medium (Sakura Finetek), sectioned into 20 μm thickness.

**Figure 3:**
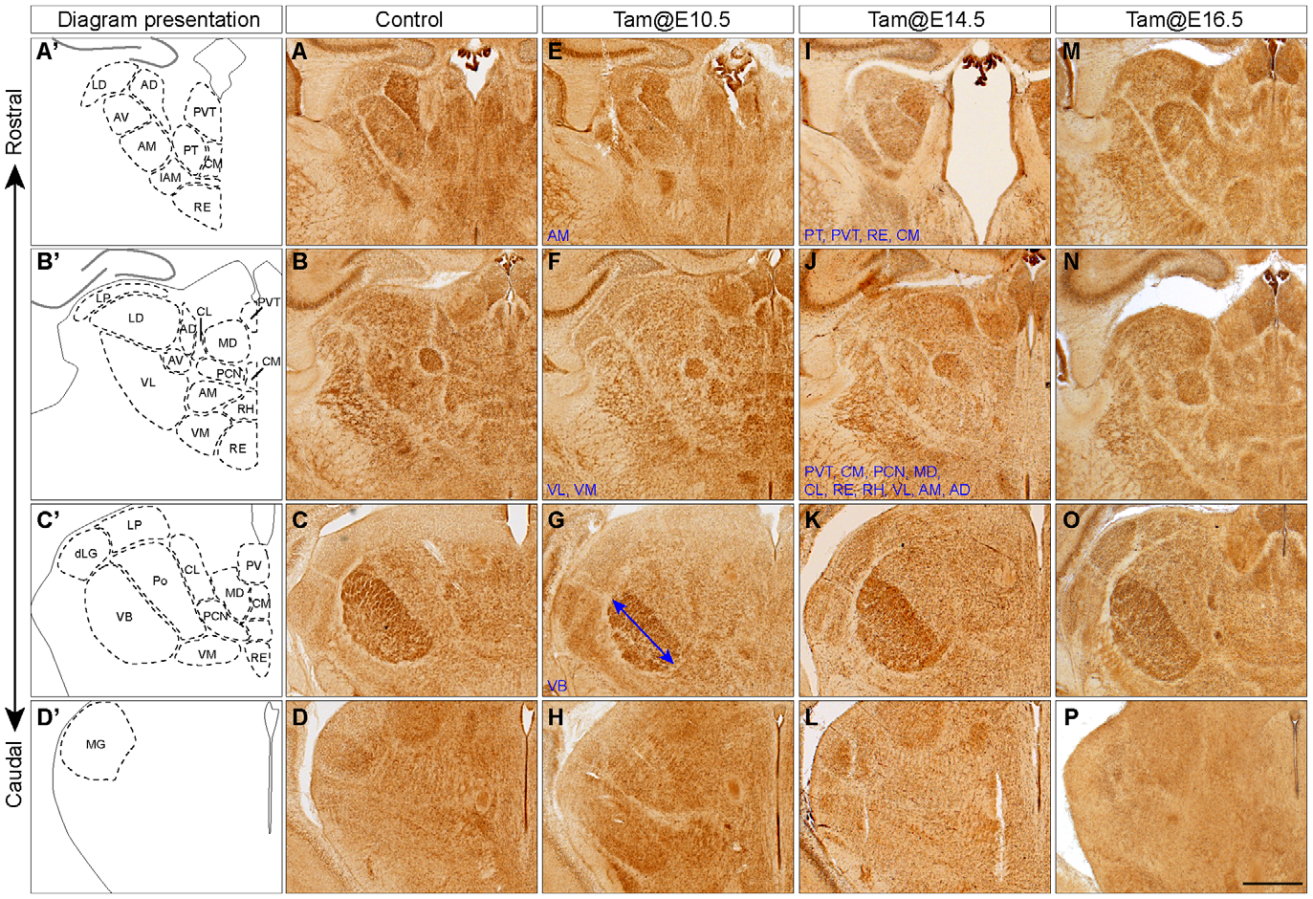
Deletion of *Gbx2* at E10.5 or E14.5, but not at E16.5, disrupts distinct thalamic nuclei. (A–P) CO histochemistry on coronal brain sections of control (A–D) and *Gbx2^creER/F^* (E–P) mice at P4 that were given tamoxifen at E10.5 (E–H), E14.5 (I–L) or E16.5 (M–P). Thalamic nuclei are outlined in control sections and the affected thalamic nuclei are listed in blue at the lower left corner. Identification and nomenclature of thalamic nuclei are based on Caviness and Frost [28] and Jones [1]. The asterisk marks the loss of tissue corresponding to the anterior-medial group of nuclei (I), and the double arrow indicates the reduced medal-lateral dimension of VB (G). Note that thalamic nuclei are indistinguishable between control and *Gbx2*-CKO mice receiving tamoxifen at E16.5. Scale bar in P: 500 µm (for A–P).

### Histochemistry, immunofluorescence, and in situ hybridization

Standard protocols were used for X-gal histochemistry, immunofluorescence, and *in situ* hybridization (ISH), as described previously [16]. For cytochrome oxidase (CO) staining, sections were stained in freshly prepared solution containing 0.5 mg/ml diaminobenzidine (Sigma), 0.2 mg/ml cytochrome C (Sigma), and 40 mg/ml sucrose in PBS as described previously [7]. The incubation period varied from 14 to 18 hours, and the reaction was terminated based on empirical observations. Detailed protocols for histochemistry, immunostaining and ISH are available in the Li Laboratory website (http://lilab.uchc.edu/Pages/Protocols.html). Primary antibodies used in the study were as follows: rabbit anti-GFP (1∶1000, Invitrogen), rat anti-GFP (1∶2000, Nacalai Tesque), rabbit anti-caspase 3 (cleaved) (1∶500, Cell Signaling Technology), rabbit anti-5-HTT (1∶1000, ImmunoStar), mouse anti-Nkx2.2 (1∶200, DSHB), rabbit anti-NPY (1∶25000, Dr. Richard Mains, University of Connecticut Health Center), rabbit anti-Sox2 (1∶250, R&D Systems), rabbit anti BLBP (1∶1000) and mouse anti-NeuN (1∶200, Millipore), and mouse anti-CC1 (1∶100, Calbiochem). Alexa fluorescent secondary antibodies (Invitrogen) were used.

**Figure 4:**
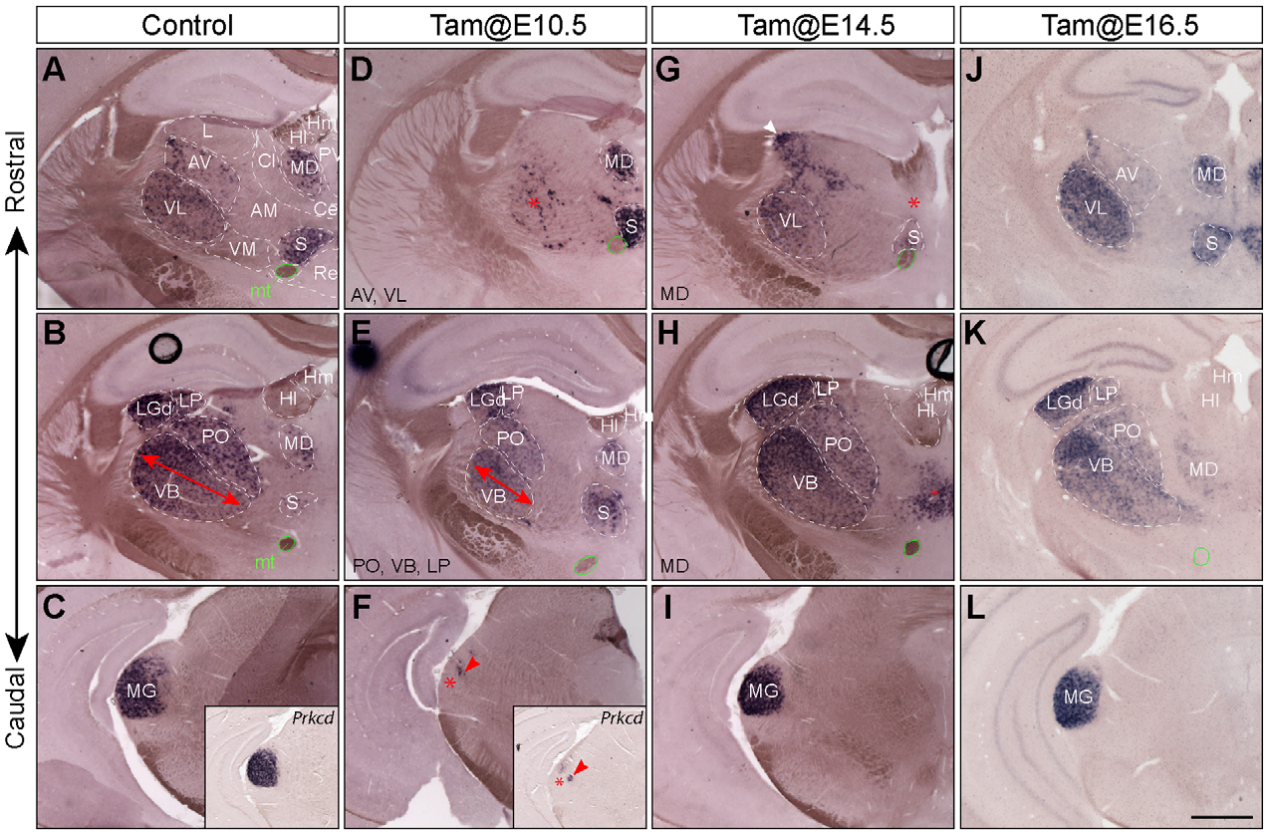
Altered expression of thalamic markers in P21 thalamus following temporal deletion of *Gbx2*. (A–L) ISH for *Tnnt1* on coronal sections of P21control (A–C) and *Gbx2^creER/F^* brains that was given tamoxifen at E10.5 (D–F), E14.5 (G–I) and E16.5 (J–L), respectively. Insets in (C) and (F) show ISH for *Prkcd*. The affected nuclei are listed in the lower left corner or indicated by an asterisk. The double arrow indicates the reduced size of VB; red arrowheads show residual *Tnnt1^+^* and *Prkcd^+^* cells in the presumptive MG nucleus; the white arrowhead indicates LP in G (this section corresponds a slightly more caudal position than those in A, D, and J). Abbreviations: mt, mamillothalamic tract; S, submedial nucleus. Scale bar in L: 500 µm (for A–L).

All phenotypes reported were consistently observed in at least three *Gbx2*-CKO mutants. To quantify cell death, double immunofluorescence for GFP and activated caspase 3 (Casp3) was performed on serial coronal sections of E16.5 or E18.5 brains. Thalamic sections were matched at five thalamic rostrocaudal levels per genotype. The outline of different pronuclei was identified with the help of EGFP expression from the *Gbx2^creER^* locus. The total number of Casp3-positive cells was determined for each pronucleus from the same number of matched sections between control and mutant brains. At least three control or three *Gbx2*-CKO embryos were analyzed to determine the average number of dead cells. Statistical difference was determined by Student's *t*-test using Prism (GraphPad Software).

**Figure 5:**
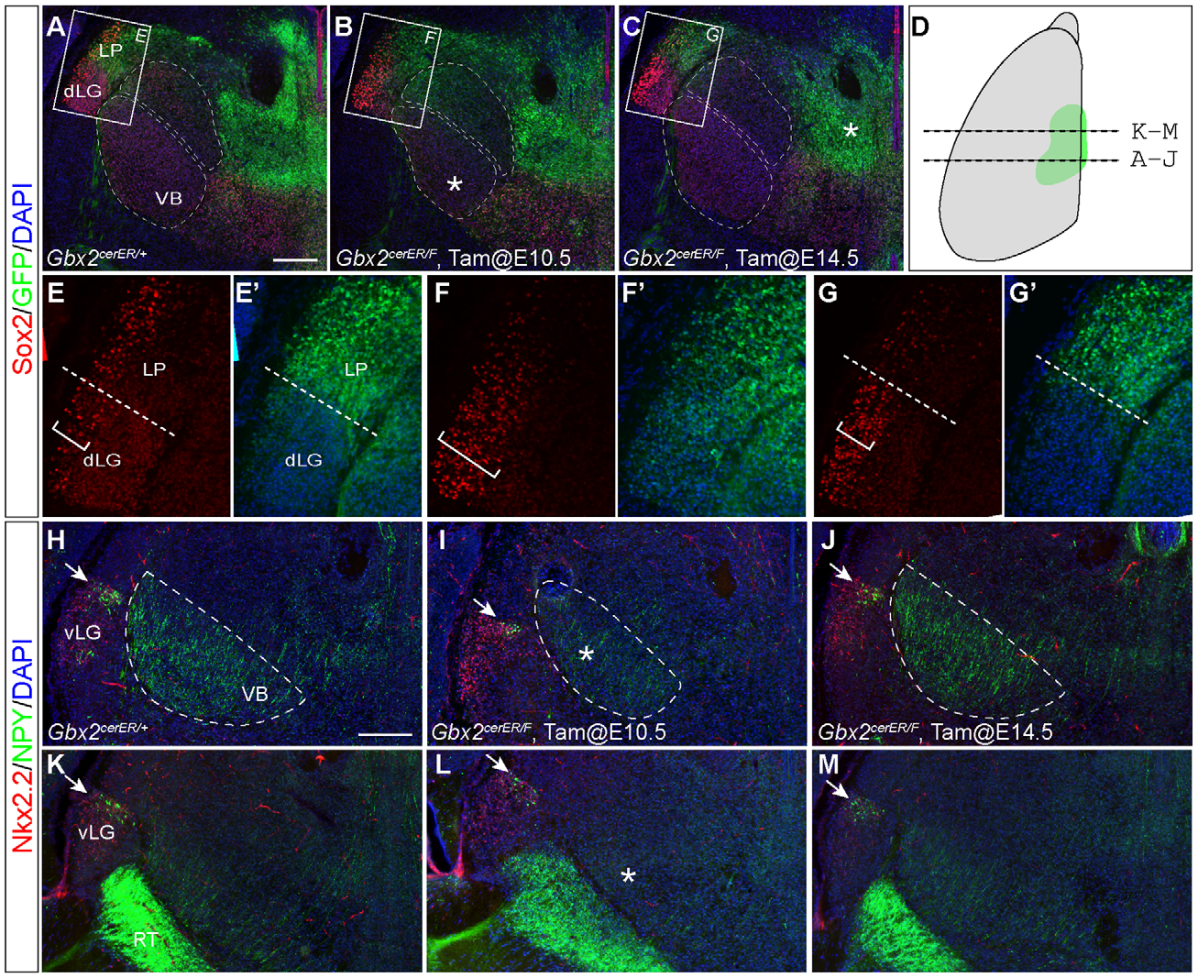
The VB and dLG nuclei are disrupted when *Gbx2* is deleted at E10.5, but not at E14.5. (A–C) Double immunofluorescence for Sox2 and GFP, which reflects the transcription of the *Gbx2* locus, on coronal sections of E18.5 brains of indicated genotypes. Boxed areas are magnified in E–G'. The asterisk indicates increased GFP expression in MD, and reduction in size of the VB; the dashed lines mark the border between Sox2 and GFP corresponding to the dLG and LP border; the brackets indicate the width of a band of strong Sox2-expressing cells along the lateral side of dLG and LP. (D) A schematic diagram indicates the position and the plane of sections shown in the figure. (H–M) Immunofluorescence for Nkx2.2 and NPY on coronal sections of E18.5 brains of indicated genotypes. Asterisks indicate the reduction of NPY+ neurites and the smaller VB in I; arrows mark NPY+ cells in the IL. Scale bars: A (A–C) 200 µm; E (for E–G'') 50 µm.

## Results

### Gbx2-expressing cells exclusively give rise to thalamic neurons

We have previously shown that *Gbx2* is mainly expressed in postmitotic cells in the thalamus [6]. To determine the neural lineage derived from *Gbx2*-expressing cells, we performed genetic inducible fate mapping in *Gbx2^creER/+^; R26R^YFP/+^* mice to permanently label *Gbx2*-expressing cells in between E10.5 and E16.5. The identity of the fate-mapped cells was examined by markers for neurons (NeuN), oligodendrocytes (CC1 and BLBP), and astrocytes (GFAP) at postnatal stages. At postnatal day (P) 21, all descendants of *Gbx2*-expressing cells that were labeled at E10.5, E14.5 or E16.5 were positive for NeuN, but negative for CC1 or GFAP (Fig. 1A–F and data not shown). There were many BLBP^+^ cells in the thalamus at P21 (Fig. 1G–I). These BLBP^+^ cells were negative for NeuN, CC1, or GFAP, suggesting that these BLBP^+^ cells are probably glial precursor cells (Fig. 1H, I, and data not shown). Importantly, none of the *Gbx2*-derived cells were positive for BLBP (Fig. 1G and data not shown). Therefore, the *Gbx2* lineage exclusively gives rise to neurons but not glia in the thalamus.

**Figure 6:**
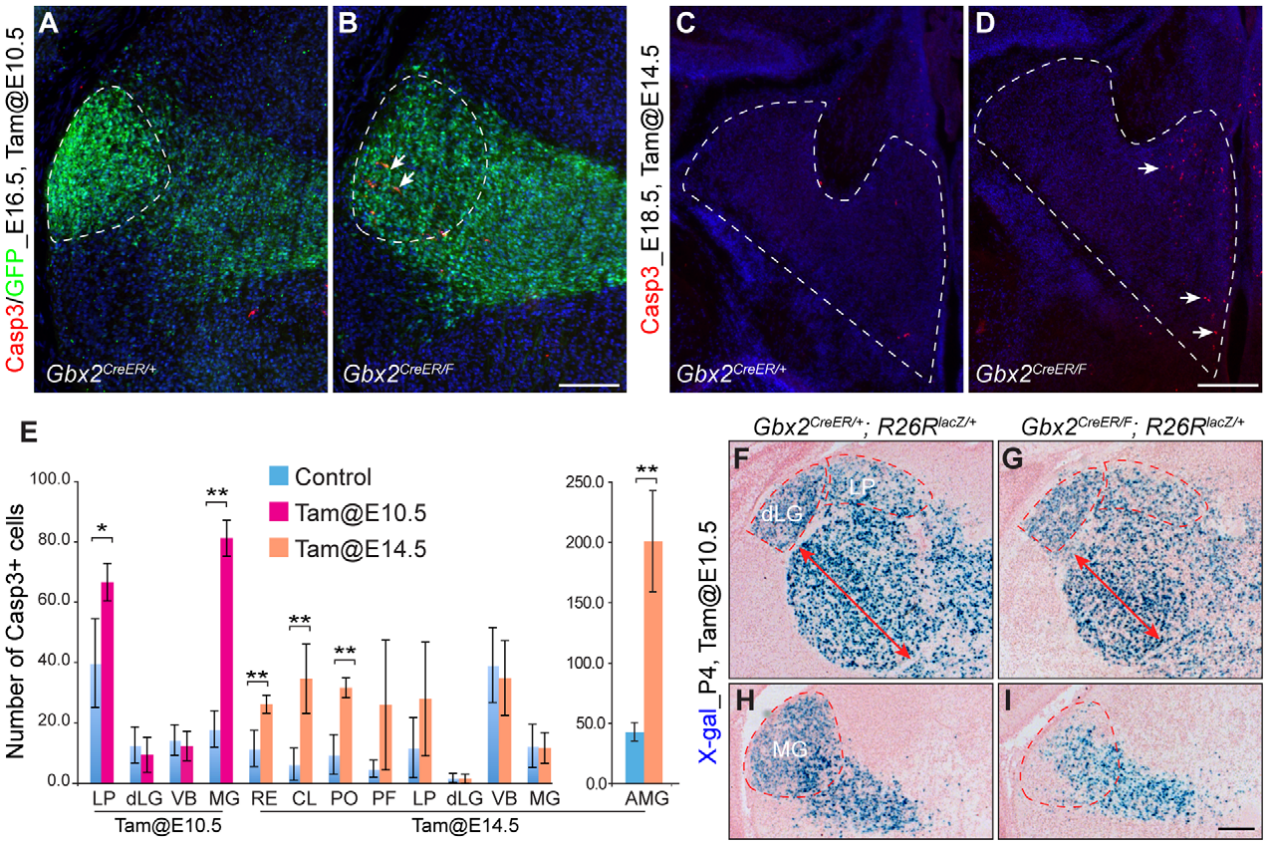
*Gbx2* is transiently required for cell survival for a subset of thalamic nuclei. (A–D) Immunofluorescence of activated Casp3 on coronal sections of the thalamus of indicated genotypes. GFP signals in A and B were derived from the *Gbx2^cereER^* allele. MG (A and B) and the dorsomedial thalamus (C and D) are outlined by the dashed lines. Arrows indicate apoptotic cells. (E) Histogram representation of the average number of apoptotic cells in different thalamic nuclei. Error bars indicate standard deviations. Asterisks * and ** indicate *p*< 0.05 and *p*<0.001, respectively (Student's *t*-test). (F–I) X-gal histochemistry on coronal brain sections of P4 mice that were given tamoxifen at E10.5. The double arrow indicates reduced size of VB in mutants. Scale bars: B (for A–B) 100 µm; D (for C–D) 200 µm; I (F–I) 250 µm.

### Removing Gbx2 at different embryonic stages disrupts distinct sets of thalamic nuclei

By fate-mapping *Gbx2*-expressing cells between E9.5 and E15.5, we have previously identified five groups of thalamic nuclei (Fig. 2A) [6]. The postmitotic neurons of principal relay nuclei, such as the dLG, VB, LP and MG nuclei, express *Gbx2* by E10.5 (Fig. 1A and Table I for abbreviations). The expression of *Gbx2* is quickly downregulated in dLG and VB (designated as group I nuclei), yet persists in LP and MG (group II). The second wave of *Gbx2*-expressing cells (E10.5–E13.5) gives rise to many association nuclei, such as the AV, CL, MD, PCN, CE, and relay nuclei, LD, VL, and VM (Fig. 1A). Among these nuclei, *Gbx2* expression persists in MD and CE, PCN and CL (group III) in postnatal stages, but is downregulated in LD and VM (group IV) after E14.5. The last wave of *Gbx2*-expressing cells (E15.5) gives rise to many anterior-medial nuclei, such as PVT, PT, RE, and AM (Fig. 1A), and *Gbx2* expression in these nuclei persists in postnatal stages [10].

**Figure 7:**
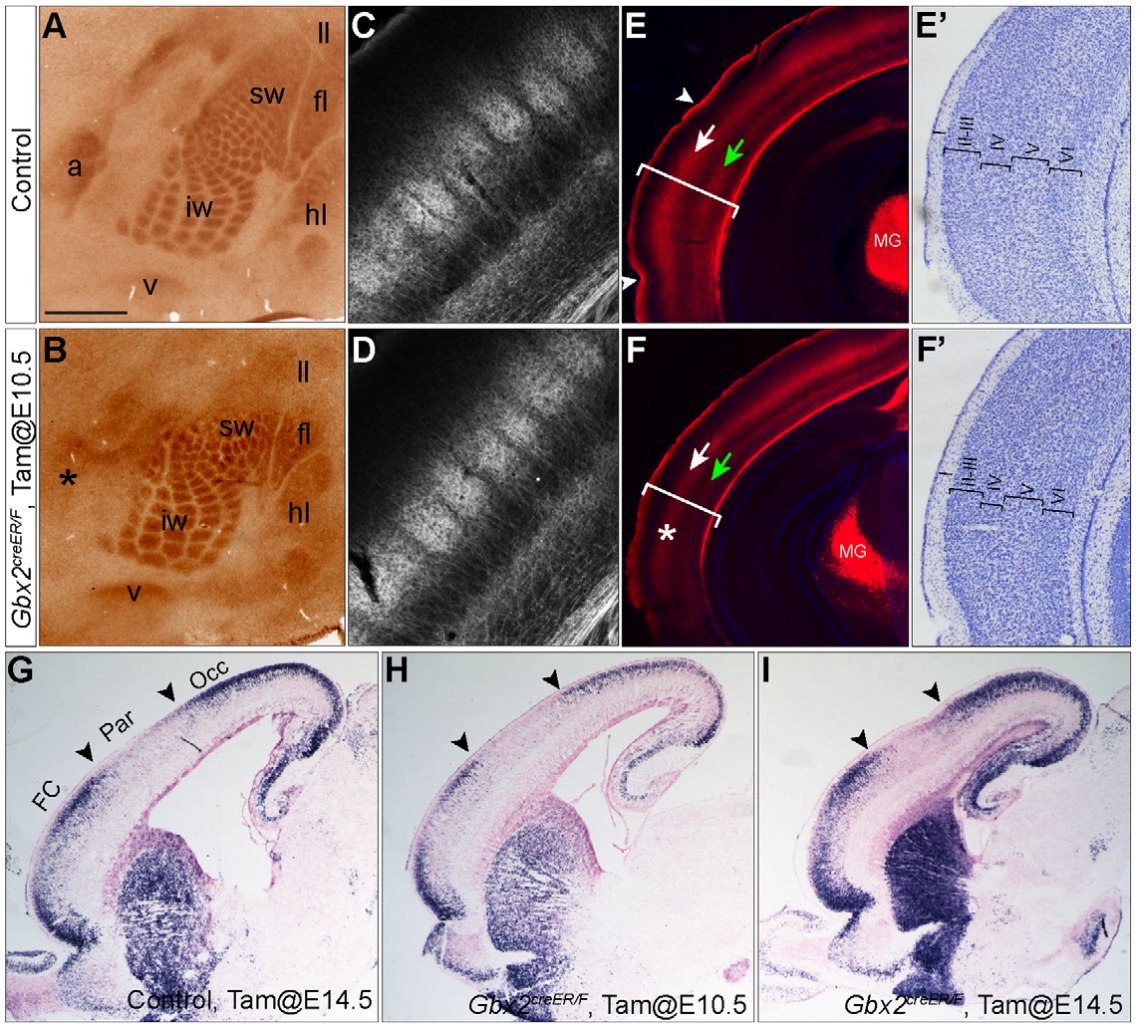
Parcellation of the cerebral cortex is unaffected due to the disruption of thalamic nuclei. (A–B) Immunohistochemistry for 5-HTT on tangential sections of P7 brains of control and *Gbx2*-CKO mice that received tamoxifen at E10.5. The 5-HTT immunohistochemistry reveals the body map in the cortex: a, auditory; v, visual; fl, florelimeb; hl, hindlimb; ll, lower lip; t, trunk; lw, large whiskers; sw, small whiskers [21]. (C–F) Red fluorescence (tdTomato) on coronal sections of P21 brains of *Gbx2^creER/+^; R26R^RFP/+^* (C and E) and *Gbx2^creER/F^; R26R^RFP/+^* (D and F) mice that were given tamoxifen at E10.5. The primary auditory area is indicated by arrowheads and cortical layers IV and VI were marked by white and green arrows, respectively. Note that the thickness (bracket) of the auditory cortex is reduced in *Gbx2*-CKO mutants. (E'–F') Nissl histology of the auditory cortex of control (E') and Gbx2-CKO mice (F'). (G–I) ISH for *Lmo4* on sagittal sections of E18.5 control and *Gbx2*-CKO mutants that received tamoxifen at E10.5 or E14.5 as indicated. Black arrowheads demarcate the borders of the somatosensory area. Abbreviations: FC frontal; Occ, occipital; Par, parietal. Scale bar in A: 250 µm (A–B); 154 µm (for C–D); 307 µm (for E–F); 189 µm (for G–I).

To investigate whether the different onset and duration of *Gbx2* expression controlled the formation of thalamic nuclei, we deleted *Gbx2* at different stages using a “self-deletion” approach in compound heterozygous *Gbx2^creER/F^* embryos by activating creER through administering tamoxifen at E9.5, E10.5, E12.5, E14.5, and E16.5 (Fig. 2A) [6], [12]. The extent of recombination and the resulting phenotype were variable when tamoxifen was administered at E9.5 and E12.5 probably because *Gbx2* expression is about initiated at E9.5 and undergoes a major transition from E10.5 to E14.5 (data not shown). Therefore, we mainly characterized the phenotype of *Gbx2*-CKO mice that were given tamoxifen at E10.5, E14.5, or E16.5 in the current study. To examine the specific deletion of *Gbx2*, we performed ISH using a *Gbx2* probe corresponding to *Gbx2* exon II that was deleted by creER-mediated recombination. When tamoxifen was given at E10.5, E14.5, or E16.5, *Gbx2* transcripts were mostly absent in the most caudal and lateral region of the thalamus, presumably the MG in *Gbx2^creER/F^* embryos (Fig. 2C–H). However, *Gbx2* mRNA was present in the anterior and medial region of the thalamus in *Gbx2^creER/F^* embryos that received tamoxifen at E10.5. By contrast, *Gbx2* transcripts were mostly abolished or reduced in the anterior-medial part of the thalamus, as well as the MG, in *Gbx2^creER/F^* embryos that received tamoxifen at E14.5 or E16.5 (Fig. 2I–N). Therefore, the self-deletion approach results in efficient and selective deletion of *Gbx2* in different regions of the developing thalamus.

*Gbx2^creER/F^* mice that received tamoxifen at E10.5 or later stages were viable and grossly normal. To examine the formation of thalamic nuclei, we performed CO histochemistry of *Gbx2-*CKO brain at P4 when thalamic nuclei were readily discernable (Fig. 3A–D). When *Gbx2* was deleted around E10.5, MG was mostly missing, and VB appeared smaller (Fig. 3G and H). The borders among group III nuclei (e.g. MD, Cl, and Ce) became less prominent, suggesting abnormal differentiation and/or aggregation of these nuclei (Fig. 3E and F). Although administration of tamoxifen at E14.5 resulted in efficient removal of *Gbx2* transcripts in the caudal and lateral thalamus (Fig. 2I), the MG and VB nuclei were mostly indistinguishable between control and the *Gbx2*-CKO mice (Fig. 3J–L). By contrast, deleting *Gbx2* at E14.5 disrupted group III nuclei, such as MD, Cl, and Ce (Fig. 3J). Furthermore, tissue corresponding to many group V nuclei (e.g. PT, PVT, and RE) was mostly missing following inactivation of *Gbx2* at E14.5 (Fig. 3I). In contrast to malformation of various thalamic nuclei due to *Gbx2* deletion at earlier stages, the CO histochemistry of the thalamus in *Gbx2*-CKO mice that received tamoxifen at E16.5 was indistinguishable from that of control mice (Fig. 3I–L).

To further characterize the thalamic nuclei affected by the loss of *Gbx2*, we examined the transcripts of *Tnnt1* (*Troponin T type 1)* and *Prkcd* (*Protein kinase C delta*), which displayed distinct levels of expression in different thalamic nuclei and thus demarcated a subset of thalamic nuclei at P21 (Fig. 4A–C). In agreement with the CO histochemistry, deletion of *Gbx2* at E10.5, but not at E14.5 or E16.5, resulted in mostly absence of the MG nucleus with only a few clusters of remnant *Tnnt1* ^+^ or *Prkcd^+^* cells in the presumptive MG area (Fig. 4E, F, H, and I). Furthermore, the VB, VL, and AV nuclei were noticeably smaller in *Gbx2^creER/F^* mice that received tamoxifen at E10.5 (Fig. 4E). In control mice, *Tnnt1* was expressed in MD (Fig. 4A and B). When *Gbx2* was deleted at E14.5, but not E10.5 nor E16.5, *Tnnt1* ^+^ cells in the presumptive MD were mostly missing, in support of the notion that group III nuclei were particularly sensitive to the deletion of *Gbx2* at E14.5 (Fig. 4G and H). No changes in the expression of *Tnnt1* and *Pcp4* were found in the thalamus of *Gbx2^creER/F^* mice given tamoxifen at E16.5 (Fig. 4J–L and data not shown), demonstrating that *Gbx2* is no longer required for thalamic histogenesis by E16.5.

In summary, deletion of *Gbx2* at different stages disrupts the formation of distinct thalamic nuclei. Interestingly, the MG formation is unaffected when *Gbx2* is deleted after E14.5 despite the persistent *Gbx2* expression in this nucleus. Furthermore, deleting *Gbx2* at E16.5 has no noticeable effect on the thalamus even though many group III and V nuclei that depend on *Gbx2* function at E14.5 continue expressing *Gbx2* after birth. These findings demonstrate that *Gbx2* is mainly required in a narrow time window immediately after the onset of its transcription for the development of thalamic nuclei.

### The initial expression of Gbx2 is essential for all principal thalamic nuclei

We have previously shown that all principal thalamic nuclei are generated from thalamic postmitotic neurons that express *Gbx2* between E9.5 and E10.5 [6]. Unexpectedly, no obvious defect in the dLG and LP nuclei was detected by CO histochemistry and *Tnnt1/Prkcd* expression analyses (Fig. 3 and 4). We thus used additional molecular markers to examine the formation of these nuclei. *Sox2* and *Gbx2* are differentially expressed in different sets of thalamic nuclei [17]. We performed double immunofluorescence for Sox2 and GFP. The latter was expressed from the *Gbx2^creER^* allele and thus mimicked the endogenous *Gbx2* expression [6]. Robust GFP expression was detected in LP, CL and PV, and a lower level of GFP expression was observed in MD nucleus in control *Gbx2^creER/+^* embryos that received tamoxifen at E10.5 (Fig. 5A). GFP was mostly absent from dLG while its expression was maintained in LP (Fig. 5A and E'). Complementary to GFP expression, Sox2 was present in VB, dLG, and PO but absent from LP, except for a strip of strong Sox2-expressing cells along the lateral side of dLG and LP (Fig. 5A and E). In *Gbx2^creER/F^* embryos that received tamoxifen at E10.5, the conspicuous dLG/LP border defined by the common Sox2/GFP border was lost with many GFP^+^ cells present in dLG (Fig. 4B, F and F'). The low level of Sox2 expression in dLG was mostly missing, whereas the strip of strong Sox2^+^ domain along the lateral side of dLG and LP became much wider (Fig. 5B and F). In agreement with CO and marker analyses described above, deleting *Gbx2* at E14.5 had little effect on the expression of Sox2 and GFP in dLG and LP nuclei (Fig. 5G and G'). Nevertheless, in these embryos, abnormal GFP expression was found in the presumptive MD, suggesting a specific requirement of *Gbx2* for the development of the MD nucleus at E14.5 (Fig. 5C, G and G'). These data demonstrate that deletion of *Gbx2* at E10.5, but not E14.5, disrupts the differentiation of dLG and LP.

It was recently shown that a narrow band of cells between the zona limitans intrathalamica (ZLI) and the thalamus gives rise to the ventral lateral geniculate body (vLG) and intergeniculate leaflet (IL) nuclei [18]. Enhancing Fgf8 or Shh signaling near the ZLI results in enlarged vLG and IL and a shrunken VB [17], [19], a phenotype similar to that found in *Gbx2^creER/F^* embryos that received tamoxifen at E10.5. Therefore, we examined the formation of IL, vLG and reticular (RT) nucleus, which are juxtaposed with dLG and VB, using double immunofluorescence for neural peptide Y (NPY) and Nkx2.2 [18]. NPY was expressed in IL and RT, and Nkx2.2 was expressed in vLG (Fig. 5H and K). The expression pattern of NPY and Nkx2.2 was unchanged when *Gbx2* was deleted at E10.5 or E14.5, demonstrating that loss of *Gbx2* does not affect the formation of the IL, RT and vLG nuclei (Fig. 5I, J, L, and M). Interestingly, NPY+ neurites, which are presumably derived from RT neurons, were greatly reduced in VB of *Gbx2^creER/F^* mice that were given tamoxifen at E10.5 (Fig. 5H–M), suggesting that *Gbx2* expression is crucial for the differentiation and the innervation by NPY neurites in the VB nucleus.

Altogether, our results demonstrate that the expression of *Gbx2* around E10.5 is important for the differentiation of all principal thalamic nuclei in agreement with the predominant contribution of the initial *Gbx2* expressing cells to these nuclei.

### Gbx2 is differentially required for the survival of neurons in distinct thalamic nuclei

It has been previously shown increased apoptosis in the thalamus in *Gbx2*-null mutants at E18.5, demonstrating that *Gbx2* is required for thalamic neuron survival [9]. However, the identity of thalamic nuclei that are dependent on Gbx2 for cell survival has not been defined. To this end, we examined the expression of activated caspase 3 (Casp3), which marks cells undergoing apoptosis, in the thalamus following inactivation of *Gbx2* at different embryonic stages. No noticeable change was found in the number of Casp3^+^ cells in the thalamus of *Gbx2^creER/F^* embryos in 48 h after tamoxifen exposure at E10.5 or E14.5 (data not shown). An increase of Casp3^+^ cells was first detected in the lateral-caudal region of the thalamus in *Gbx2^creER/F^* embryos at E14.5 following tamoxifen administration at E10.5 (data not shown). As it is difficult to identify the affected nuclei at E14.5, we focused our analysis on E16.5 when some thalamic pronuclear masses begun to be discernable. Significantly increased Casp3^+^ cells were restricted to the presumptive LP and MG nuclei following deletion of *Gbx2* at E10.5 (Fig. 6A, B, and E). By contrast, Casp3 expression in the presumptive dLG and VB region was indistinguishable between controls and mutants (Fig. 6E). When *Gbx2^creER/F^* embryos were given tamoxifen at E14.5, significant increase in the number of Casp3^+^ cells was found in RE, CL, PO, MD, and nuclei in the anterior-medial groups (AMG) at E18.5 (Fig. 6C, D and E).

To confirm that cell death accounts for the loss of MG and LP, we followed the fate of cells that undergone creER-mediated recombination in *Gbx2^creER/F^* mice carrying the cre reporter *R26R^lacZ^*. Although X-gal expression is not necessarily indicative for *Gbx2*-deficiency at the cellar level because the *Gbx2*-floxed allele and the reporter allele may have different recombination efficiency, the recombination at these two loci should be in agreement with each other in a particular cell cohort. In P4 *Gbx2^creER/F^; R26R^+/lacZ^* mice that were given tamoxifen at E10.5, X-gal histochemistry revealed that the density of ß-gal^+^ cells was noticeably reduced in the LP and MG, but was unchanged in the dLG and VB nuclei (Fig. 6F–I). Although not all marked cells were necessary mutant, the specific loss of X-gal positive cells in the MG, together with the result of Casp3 analyses, show that *Gbx2* is essential for neuron survival in MG and LP, but not in dLG and VB nuclei.

In summary, although loss of *Gbx2* disrupts formation of the majority of thalamic nuclei, only a subset of thalamic nuclei, such as the MG, L, RE, CL, and PO, depends on *Gbx2* for cell survival.

### Parcellation of the cortex is mostly intact in Gbx2-CKO mutants

The principal thalamic nuclei project to specific sensory cortical areas. We next examined the innervation of thalamocortical axons (TCAs) in the sensory cortical regions. TCAs innervating the cerebral cortex in neonatal mice transiently store serotonin (5-HT) in synaptic vesicles by expressing serotonin transporter (5-HTT) [20]. Therefore, immunohistochemistry for 5-HT or 5-HTT reveals the body map in layer IV of the cortex in P7 mice [21] (Fig. 7A and data not shown). In agreement with the loss of the MG nuclei, which innervate the auditory cortex (A1), 5-HTT immunoreactivity in the A1 area was greatly reduced in *Gbx2*-CKO mutants that were given tamoxifen at E10.5 (Fig. 7B). Interestingly, although the VB was affected by *Gbx2* deletion at E10.5 (Fig. 3G, 4E, 5B and I), the somatosensory barrels were present in *Gbx2*-CKO mutants (Fig. 7B). Examination by CO histochemistry also showed normal somatosensory barrels in *Gbx2*-CKO mutants that received tamoxifen at E10.5 (data not shown). To further examine the barrels in the cortex, we used the cre report *R26R^RFP^* that expressed robust tdTomato from the *ROSA* locus [15], and permanently labeled TCAs following *Gbx2-creER* mediated recombination. As expected, TCAs were mostly detected in cortical layers IV and VI in *Gbx2^creER/+^; R26R^+/RFP^* and *Gbx2^creER/F^; R26R^+/RFP^* mice that received tamoxifen at E10.5 (Fig. 7C–F). In the somatosensory cortex, RFP^+^ neurites clearly demarcated the individual barrels in *Gbx2*-CKO mutants as well as in the control (Fig. 7C and D). In agreement with the specific cell death in MG nucleus, RFP^+^ neurons were significantly reduced in MG nuclei and RFP^+^ neurites were noticeable reduced in layers IV and VI in the auditory cortex in *Gbx2*-CKO mice (Fig. 7E and F). Despite the decrease in the overal thickness, the layered structure of the typical auditory cortex was maintained in *Gbx2*-CKO mice (Fig. 7E'–F'). To examine the parcellation of the cerebral cortex, we examined the expression of *Lmo4* and *Lmo3*, which demarcated the key subdivision in the cerebral cortex [22], on a whole series of coronal and sagittal sections of E18.5 brains (Fig. 7G–I and data not shown). No noticeable changes were detected in the expression of these genes in the cortex in *Gbx2*-CKO embryos at E18.5 when *Gbx2* was removed at E10.5 or E14.5.

In summary, we found that deletion of *Gbx2* at E10.5 results in greatly reduced TCA projections from the MG and alterations in the auditory cortex. However, the parcellation and the subsequent formation of other body maps, including the sensory barrels, are mostly unaffected by the loss of *Gbx2* in the thalamus.

## Discussion

Expression and fate mapping studies suggested that *Gbx2* might play an important role in the specification and differentiation of thalamic nuclei [6], [10]. Indeed, thalamic nuclei, particularly those in the medial, central, and dorsal groups, are severely disrupted in *Gbx2* global KO embryos at E18.5 [5], [9]. However, the severe defects in thalamic histogenesis and neonatal lethality have precluded the analysis of *Gbx2* function in any specific thalamic nucleus. Here, we have examined the temporal requirements of *Gbx2* and have identified an essential but transient *Gbx2* function in the differentiation and/or survival of neurons in different thalamic nuclei.

As arrays of glial cells were previously seen clustered at the boundary of different groups of neurons, it has been proposed that glia may contribute to nuclear formation in the brain [23]. Indeed, we found that the anterior limit of the thalamus was clearly demarcated by a large amount of GFAP^+^ cells in the IGL and zona limitans intrathalamica (Fig. 1D and data not shown). Compared to the neighboring structures, we found that the matured thalamus contained only a low number of GFAP^+^ astrocytes (Fig. 1 and data not shown). By contrast, there were a large number of BLBP^+^ cells in the thalamus (Fig. 1). These BLBP^+^ cells were negative for NeuN, CC1, and GFAP, and their precise identity remains to be determined. However, we did not detect any regional differences in the distribution of CC1^+^, GFAP^+^ or BLBP^+^ cells within the thalamus. Furthermore, we showed that all the descendants of *Gbx2* expressing cells that were labeled at E10.5, E14.5 or E16.5 were positive for NeuN but not CC1, GFAP, or BLBP (Fig. 1 and data not shown), demonstrating an exclusive contribution of the *Gbx2* lineage to thalamic neurons. Therefore, it is likely that *Gbx2* mainly acts on neurons but not glia during the differentiation of thalamic nucleus.

We have previously shown that the onset of *Gbx2* is generally associated with the commencement of neurogenesis of thalamic neurons [6]. Interestingly, although the entire thalamic nuclear complex is derived from the *Gbx2*-lineage, different groups of nuclei display distinctive temporal expression patterns of *Gbx2* [6]. Therefore, the dynamic expression of *Gbx2* may act as a determinant for nucleus-specific neurons. Alternatively, *Gbx2* is important for assigning a general thalamic identity, which is essential for the subsequent differentiation of the thalamic nucleus. In the current study, we showed that inactivation of *Gbx2* disrupted formation of the majority of thalamic nuclei, confirming an essential role of *Gbx2* in the formation of thalamic nuclei. Importantly, we demonstrated that deleting *Gbx2* at E10.5 or E14.5 mostly affected the same set of nuclei that were composed of fate-mapped *Gbx2*-expressing neurons labeled at that particular stage (Table I). Furthermore, the nuclei that were specifically affected by *Gbx2* deletion at E10.5 or E14.5 were mostly unaffected when *Gbx2* was removed at E14.5 or E16.5 regardless of whether *Gbx2* expression persists in these nuclei normally. These results demonstrate that *Gbx2* plays a crucial role in the formation of various thalamic nuclei in a narrow time window after the onset of its expression. Although our molecular marker analyses were still limited due to the lack of nucleus-specific markers, we have not observed any overt change in nucleus identity in *Gbx2*-CKO mice (Fig. 4 and 5). In these mutants, the loss or reduction of particular nucleus was not associated with expansion of any neighboring nuclei. Furthermore, by combining fate mapping and *Gbx2* deletion, we showed that there were no noticeable changes in the distribution of recombined cells between *Gbx2^creER/+^; R26R^lacZ/+^* and *Gbx2^creER/F^; R26R^lacZ/+^* mice (Fig. 6F–I and data not shown). Therefore, we favor the second model in which *Gbx2* may be important for newly generated neurons to acquire a general thalamic identity immediately following neurogenesis.

We have extended the previous finding [9] by defining the thalamic nuclei that depend on *Gbx2* for neuron survival. Interestingly, although *Gbx2* was dispensable after its initial expression, inactivation of *Gbx2* resulted in cell death in groups II, III, and V nuclei that maintain *Gbx2* expression but not in groups I and IV nuclei that transiently express *Gbx2* (Table I). This bias might result from less efficient deletion of *Gbx2* in the latter groups due to transient creER expression. However, this explanation seems unlikely, since we found that the creER-mediated recombination was comparable among nuclei with either transient or prolonged *Gbx2* expression by using different cre-reporter lines (Fig. 6F and data not shown). The molecular mechanism by which Gbx2 regulates cell survival in the thalamus is currently unknown. It has been previously shown that the survival of thalamic neurons is partially controlled by trophic factors from the cerebral cortex [24]. In *Gbx2* global KO mutants, thalamocortical projections are mostly absent [5], . Conditional deletion of *Gbx2* also resulted in partial loss of thalamocortical axons (Li and Li, unpublished data). Therefore, further studies will determine whether the requirement of *Gbx2* for cell survival in a particular nucleus is related to its dependence on *Gbx2* to establish axonal connection with the cortex.

Despite the absence of abnormal apoptosis, some nuclei, such as VB, VL, and AV, were decreased in size following *Gbx2* deletion at E10.5 (Fig. 3, 4 and 6). This suggests that, in additional to increased cell death, other defects, such as abnormal neurogenesis or altered neuronal migration, may also contribute to the abnormal development of thalamic nuclei in *Gbx2*-CKO mutants. Although defects were detected in most thalamic nuclei by CO histology and marker analyses, some nuclei appeared unaffected (e.g. AD) or only displayed only subtle defects by *Gbx2* deletion at E10.5 or E14.5 (Table I). We cannot rule out that defects may be present in these seeming normal nuclei following *Gbx2* deletion at E10.5, E14.5, or even E16.5.

### Concluding remark

The temporal order of neurogenesis plays an important role in not only the generation of cell type diversity, but also connection diversity of projecting neurons [25], [26]. Interestingly, in both rodents and primates, the differentiation of thalamic neurons and their connectivity closely relate to the temporal birth order: the early-born neurons tend to form the lateral-posterior nuclei and project to the occipital cortex, while late-born neurons tend to contribute to the medial-anterior nuclei and project to the frontal cortex [27]. We have previously shown that the onset of *Gbx2* expression is closely associated with the cell cycle exit of thalamic neuron precursors [6]. In the current study, we found that the initial expression of *Gbx2* plays a crucial role in the development of thalamic nuclei. Therefore, the dynamic expression of *Gbx2* may act as an important determinant in coupling with other developmental program to generate nucleus-specific neurons. Further studies of the mechanisms underlying the function of dynamic *Gbx2* expression would provide important insights into the specification and differentiation of different thalamic nuclei, and subsequently the establishment of topography of TCAs.
